# Eating attitudes of migraine patients in Turkey: a prospective multi-center study

**DOI:** 10.1186/s12883-024-03672-6

**Published:** 2024-05-29

**Authors:** Ruhsen Ocal, Basak Karakurum-Goksel, Mert Van, Ozlem Coskun, Cihad Karaaslan, Serap Ucler, Figen Gokcay, Nese Celebisoy, Hadiye Sirin, Aysin Kisabay Ak, Aysegul Seyma Saritas, Tuba Cerrahoglu Sirin, Buse Rahime Hasirci Bayir, Esme Ekizoglu, Elif Kocasoy Orhan, Derya Bayram, Nermin Tanik, Sebnem Bicakci, Vesile Ozturk, Levent Ertugrul Inan, Kubra Mehel Metin, Yasemin Eren, Babur Dora, Emel Oguz-Akarsu, Necdet Karli, Emel Ur Celik, Arife Cimen Atalar, Rabia Gokcen Gozubatik Celik, Belgin Mutluay, Elif Ilgaz Aydinlar, Pinar Yalinay Dikmen, Sencer Semercioglu, Ufuk Emre, Osman Cagin Buldukoglu, Busra Er, Bekir Burak Kilboz, Seray Ibis, Sibgetullah Yagiz, Huzeyfe Koklu, Ibrahim Kamaci, Gulshan Aliyeva, Basak Elcin Ates, Muge Mercan Kara, Fatma Zehra Altunc, Ilgin Kaya, Cagla Sisman

**Affiliations:** 1 Department of Neurology, University of Health Sciences, Antalya Training and Research Hospital, Antalya, Turkey; 2 Faculty of Medicine, Department of Neurology, Baskent University, Turgut Noyan Adana Hospital, Adana, Turkey; 3 Faculty of Medicine, Department of Neurology, Gazi University, Ankara, Turkey; 4 Faculty of Mediciene, Department of Neurology, Kirikkale University, Kirikkale, Turkey; 5 Prof. Dr. Cemil Tascioglu City Hospital, Department of Neurology, Istanbul, Turkey; 6 Faculty of Medicine, Department of Neurology, Ege University, Izmir, Turkey; 7 Faculty of Medicine, Department of Neurology, Celal Bayar University, Manisa, Turkey; 8 Department of Neurology, Sisli Hamidiye Etfal Training and Research Hospital, Istanbul, Turkey; 9 Department of Neurology, University of Health Sciences, Haydarpasa Numune Training and Research Hospital, Istanbul, Turkey; 10 Faculty of Medicine, Department of Neurology, Istanbul University, Istanbul, Turkey; 11 Department of Neurology, Adana City Training and Research Hospital, Adana, Turkey; 12 Faculty of Medicine, Department of Neurology, Yozgat Bozok University, Yozgat, Turkey; 13 Faculty of Medicine, Department of Neurology, Cukurova University, Adana, Turkey; 14 Faculty of Medicine, Department of Neurology, Dokuz Eylul University, Izmir, Turkey; 15 Department of Neurology, Ankara Training and Research Hospital, Ankara, Turkey; 16 Diskapi Training and Research Hospital, Department of Neurology, Ankara, Turkey; 17 Faculty of Medicine, Department of Neurology, Akdeniz University, Antalya, Turkey; 18 Faculty of Medicine, Department of Neurology, Uludag University, Bursa, Turkey; 19 Department of Neurology, Istanbul Kanuni Sultan Suleyman Training and Research Hospital, Istanbul, Turkey; 20 Istanbul Bakirkoy Prof Dr Osman Mental Health and Neurological Diseases Training and Research Hospital, Clinic of Neurology and Neurosurgery, Istanbul, Turkey; 21 Faculty of Medicine, Department of Neurology, Acıbadem University, Istanbul, Turkey; 22 Department of Neurology, Istanbul Training and Research Hospital, Istanbul, Turkey; 23 Department of Gastroenterology, University of Health Sciences, Antalya Training and Research Hospital, Antalya, Turkey

**Keywords:** Primary headache disorders, Migraine, Eating disorder, Pain, Depression, Anxiety, Trace amins

## Abstract

**Background:**

Migraine is a disease characterized by headache attacks. The disease is multifactorial in etiology and genetic and environmental factors play role in pathogenesis. Migraine can also be accompanied by psychiatric disorders like neurotism and obsessive compulsive disorder. Stress, hormonal changes and certain food intake can trigger attacks in migraine. Previous studies showed that eating attitudes and disorders are prevalant in patients with migraine. Eating disorders are psychiatric disorders related to abnormal eating habits. Both migraine and eating disorders are common in young women and personality profiles of these patient groups are also similar. A possible relationship which shows that migraine and eating habits are related can lead to a better understanding of disease pathogenesis and subsequently new therapeutic options on both entities. Association of migraine in relation to severity, depression and anxiety and eating habits and disorders were aimed to be investigated in this study.

**Methods:**

The study was designed as a prospective, multi-center, case control study. Twenty-one centers from Turkey was involved in the study. The gathered data was collected and evaluated at a single designated center. From a pool of 1200 migraine patients and 958 healthy control group, two groups as patient group and study group was created with PS matching method in relation to age, body-mass index, marital status and employment status. Eating Attitudes Test-26 (EAT-26), Beck’s Depression Inventory (BDI) and Beck’s Anxiety Inventory (BAI) were applied to both study groups. The data gathered was compared between two groups.

**Results:**

EAT-26 scores and the requirement for referral to a psychiatrist due to symptoms related to eating disorder were both statistically significantly higher in patient group compared to control group (*p* = 0.034 and *p* = 0.0001 respectively). Patients with migraine had higher scores in both BDI and BAI compared to control group (*p* = 0.0001 and *p* = 0.0001 respectively). Severity of pain or frequency of attacks were not found to be related to eating attitudes (r:0.09, *p* = 0.055).

**Conclusions:**

Migraine patients were found to have higher EAT-26, BDI and BAI scores along with a higher rate of referral to a psychiatrist due to symptoms. Results of the study showed that eating habits are altered in migraine patients with higher risk of eating disorders. Depression and anxiety are also found to be common amongst migraine patients.

## Introduction

Migraine is a common disorder affecting physical, mental and socioeconomical wellbeing of patients. It is the most common disorder to cause disability between women of 15 to 49 years of age, and is the second most common disabling disorder worldwide [1].

The International Classification of Headache Disorders, 3rd Edition (ICHD-3) defines migraine as a common primary headache disorder. Migraine can be divided into two major types, being with or without aura; and a third type: chronic migraine [2].

Migraine pathogenesis is multifactorial. Age, sex, genetics and environmental factors contribute to disease development. Endogenous and exogenous factors can trigger migraine attacks. Certain food types are found to trigger migraine attacks as exogenous offenders [3]. Healthy eating habits are shown to be positively effective in disease course [4]. Alcohol and coffee are triggering agents that are shown to have varying affects in different cultures on migraine attacks with both offending and protective aspects shown in studies [5, 6]. Another Mendelian study revealed coffee, cheese, alcohol and white bread unrelated to migraine; whereas corn bread, cookies and poultry were found to be related to disease course [7]. Eating habits and consumed food are important in migraine in that they are modifiable pathogenetic factors. First-line treatment of migraine is cessation of identifiable risk factors and dietary habits are disease modifying parameters in migraine. This relationship can be owed to brain-gut axis or epigenetics [8].

Psychiatric disorders are commonly seen alongside migraine. Anxiety disorders, depression, personality disorders, bipolar disorder and post-traumatic stress disorder accompany migraine in a substantial portion of patients [9]. Eating disorders (ED) are also common in migraine patients alongside depression and anxiety [10]. Pathological eating and abnormal weight-control behaviours are the backbones of EDS. Commonly known eating disorders are bulimia nervosa (BN), anorexia nervosa (AN) and binge-eating disorder with pica, rumination disorder and avoidant-restrictive food intake classified as EDs recently [11, 12].

Similarities between migraine and ED patients were shown in a review involving multiple studies. Overeating and fasting were shown to trigger migraine attacks. Relatedly, weight loss and/or lack of essential nutiritonal elements were shown to lead to headache attacks. Obsessive-compulsive disorder, perfectionalism and neuroticism were shown to increase the risks for both migraine and ED, suggesting an association between the two disease entities [12].

Interestingly, EDs and migraine share similar dysfunctions in catecholamins [13]. Plasma levels of octapamin and tyramine are elevated in both migraine and ED patients. High level of neurotransmitters are suggestive of the roles of limbic system, amygdala and hypothalamus in disease pathogenesis. All the aforementioned data supports that migraine can be a risk factor for EDs [14–16].

Treatment related factors, lifestyle changes, comorbidities and correction of exogenous offending agents are crucial in management of migraine patients [17]. Determination of modifiable risk factors helps in understanding the pathogenesis of migraine. Additionally, it would help directly in management of pain in migraine. Some triggers for migraine attacks are easily avoidable while others are unmodifiable. The end result of epidemiological and observational studies is that eating habits are important in migraine development [6, 8]. Stress management, healthy life practices including healthy eating habits and exercise are protective against migraine from taking a chronic course [18].

Cognitive behavioral therapy has been shown to be effective in non-psychiatric disorders like migraine, fibromyalgia, insomnia and chronic headache alongside EDs. Compassion-Focused Therapy (CFT) has also been shown to improve emotional control and ameliorate pain in migraine patients [19].

Management of stress and elimination of exogenous factors are therapeutical options in migraine patients who are not suitable for medical therapy due to drug intolerance or comorbidities. Migraine can be seen in any age group. Considering the continuous nature of disease and potential side effects of medications, correction of eating disorders are important in management of migraine.

With this study, we aimed to investiage the effects of eating disorders in migraine patients by utilization of Eating Attitudes Test-26 (EAT-26) [20]. EAT-26 has been verified for Turkish population [21].

## Materials and methods

The study has been designed as a multi-center, prospective, case-control study. Meetings were set beforehand with participant centers and a data sheet was created to gather and record study data. 21 centers from different parts of Turkey participated in the study. Study data involving demographic characteristics of the population were gathered and data from all participant centers were checked and recorded to SPSS software at one study center.

Study participants were volunteers with migraine diagnosis between 18 and 70 years of age, who had at least primary level of education (4 years), with the mental ability to complete surveys and understand the scope of the study, and who did not use any drugs, including migraine prophylaxis.

Individuals with a history of head trauma, smokers, pregnant or breastfeeding women, and patients with neurological, endocrine or systemic diseases other than migraine were excluded from the study.

From a pool of 1200 migraine patients and 958 healthy people, 531 participants were selected for each group with PS matching method. The control group consisted of 531 healthy volunteers between 18 and 70 years of age, who were similar with the patient group in terms of age, sex, body mass index and education level. Exclusion criteria for migraine patients listed above were also applied to the control group. In addition, individuals with a history of headache diagnosis or those who experienced headaches over the past year were also excluded from the control group. The study included 531 patients diagnosed with episodic migraine according to the 3rd edition of the International Classification of Headache Disorders (ICHD-3), 2013. Migraine diagnosis was made based up on patient history and physical examination by neurologists specialized in headache and its management from participant centers. Only episodic migraine patients were enrolled in the study since psychiatric comorbidities are more prevalent in chronic migraine patients.

EAT-26, Beck’s Depression Inventory (BDI) and Beck’s Anxiety Inventory (BAI) were applied to each participant face-to-face [20, 22, 23]. All three of the indexes used in the study were verified for Turkish population [21, 24, 25].

EAT-26 test is the most commonly used test worldwide for evaluation of eating habits. EAT-26 consists of three parts. Part A includes 7 questions regarding demographic characteristics. like weight, height and lowest and highest weight. Part A is used to give general information about the patient and is not included in scoring. Part B consists of 26 questions regarding eating habits. “Always” stands for 3 points, “usually” stands for 2 points, “often” stands for 1 point and other answers matching with rarer behavior stands for 0 point. This point system is reverse for only question 26 in this section. Each point taken here is added and the cumulative result reflects a disorder in eating habits if the total sum is equal to or above 20 points. Dissatisfaction with body morphology and psychological symptoms are shown to be related to higher scores in this section. Part C aims to evaluate eating disorders in the last six months with 5 questions on eating habits. This part is not included in calculation of test scores but a positive answer to one question in this section is an indication for referral to a psychiatrist for evaluation [20, 21].

BDI consists of 21 symptom categories with 4 answers for each question. The participant is asked to select the most suitable answer for the last week including the day the test is taken. Each question is scored from 0 to 3, and the total sum is the test result. Higher scores are correlated with depression and its severity. The results end up in one of four categories which are minimal (scores of 0 to 9), mild (scores of 10 to 16), moderate (scores of 17 to 29) and severe depression symptoms (scores of 30 to 63) [22, 24].

BAI is a self evaluation index consisting of 21 questions which are scored between 0 and 3 each. The level of discomfort and anxiety during the last week is questioned. Higher scores mean higher anxiety levels. Results are given as normal (scores of 0 to 8), low anxiety (scores of 8 to 15), moderate anxiety (scores of 16 to 25) and high anxiety (scores of 26 to 63) [23, 25].

### Statistical analysis

Categorical variables were presented as the frequency and percentage and continuous variables were presented as the mean (± SD) or median with range. Categorical variables were compared by Chi-square test or Fisher’s exact test. Continuous variables between groups were analyzed by Student’s T test for normally distributed variables and Mann-Whitney U test for non-normally distributed variables. *P* values below 0.05 were considered statistically significant. All statistical analysis were performed by SPSS 25.0.

## Results

Overall, 531 patients with episodic migraine were included in the study with matching controls. 121 (22.7%) of the patients had migraine with aura and 410 patients (77.3%) of the patients had migraine without aura. Mean number of attacks per month was 5 attacks per month in the patient group and mean level of pain according to Visual Analogue Scale (VAS) was 7. Among patients with migraine enrolled in the study, 407 (76.6%) had nausea, 125 (23.5%) had vomiting, 403 (79.4%) had photophobia and 380 (71.5%) had phonophobia during attack periods. Pain during attacks were unilateral in 320 (60.2%) of patients.

Age, education level and body-mass index (BMI) parameters were similar between patient and control groups. Mean age of the control group was 27.8 ± 7.5 years, with a median of 25 years of age (range 18–69). Mean age of the patient group was 28.7 ± 7.7 years, with a median of 27 years of age (range 18–70). Mean BMI of the control group was 24.7 ± 5.9 kg/m^2^, with a median of 24 kg/m^2^ (range 11–45). Mean BMI of the patient group was 24.3 ± 4.4 kg/m^2^, with a median of 24 kg/m^2^ (range 12–43). Mean education level of control group was 15.8 ± 2.9 years, with a median of 16 years (range 5–30). Mean education level of control group was 13.9 ± 3.4 years, with a median of 14 years (range 5–26). Age, BMI and education level comparisons of study population are given in Table 1.

**Table 1:** Age, BMI and education level comparisons of study population

	Control group (*n* = 531)		Patient group (*n* = 531)		
	Mean ± SD	Median (range)	Mean ± SD	Median (range)	p
Age (years)	27.8 ± 7.5	25 (18–69)	28.7 ± 7.7	27 (18–70)	0.127
BMI (kg/m^2^)	24.7 ± 5.9	24 (11–45)	24.3 ± 4.4	24 (12–43)	0.155
Education level (years)	15.8 ± 2.9	16 (5–30)	13.9 ± 3.4	14 (5-26)	0.050

BMI: Body-mass index, SD: Standart deviation

Females were higher in percentage for both patient and control groups, as expected per literature (69.5% and 60.8% respectively). Occupational status and marital status did not differ significantly between control group and patient group. 302 participants (56.9%) in control group were occupied full-time while 201 (37.9%) were unemployed. 298 patients (56.1) in patient group were occupied full-time while 218 (41.0%) were unemployed. Marital status of the study population were also similar between two groups. 173 participants (32.6%) were married in control group whereas 208 patients (39.2%) were married in the patients group. Single participants were in majority for both groups, with percentages of 65.9% for the control group and 58.8% for the patient group. Sex distribution, occupational status and marital status of the study population are given in Table 2.

**Table 2:** Sex distribution, occupational status and marital status of study population

	Control group (*n* = 531)		Patient group (*n* = 531)		
	n	%	n	%	p
*Sex*	531		531		
Female	323	60.8	369	69.5	0.004
Male	208	39.2	162	30.5	
*Occupation*	531		531		
Full-time	302	56.9	298	56.1	0.131
Part-time	16	3.0	15	2.8	
Unemployed	201	37.9	218	41.0	
*Marital status*	531		531		
Married	173	32.6	208	39.2	0.52
Single	350	65.9	312	58.8	
Divorced	8	1.5	11	2.1	

Indexes applied in the study were compared between two groups. 299 participants (56.1%) in control group had minimal scores in BDI whereas 204 patients (38.4%) in patient group had minimal scores in BDI. Conversely, control group had a lower percentage of participants with severe BDI scores compared to patient group. 18 participants (3.4%) in control group had severe scores in BDI whereas 31 patients (5.8%) in patient group had severe scores in BDI.

In terms of BAI, 291 participants (54.8%) in control group had normal scores in BAI whereas 178 patients (33.5%) in patient group had normal scores in BAI. Conversely, control group had a lower percentage of participants with severe BAI scores compared to patient group. 38 participants (7.2%) in control group had severe scores in BAI whereas 75 patients (14.1%) in patient group had severe scores in BAI.

Migraine patients were found to have higher BDI and BAI scores compared to control group and this difference was statistically significant (Table 3).

**Table 3:** BDI and BAI results of study population

	Control group (*n* = 531)		Patient group (*n* = 531)		
	n	%	n	%	p
BDI	531		531		
Minimal	299	56.1	204	38.4	0.0001
Mild	141	26.6	171	32.2	
Moderate	73	13.7	125	23.5	
Severe	18	3.4	31	5.8	
BAI	531		531		
Normal	291	54.8	178	33.5	0.0001
Mild	129	24.3	168	31.6	
Moderate	73	13.7	110	20.7	
Severe	38	7.2	75	14.1	

BDI: Beck’s Depression Inventory, BAI: Beck’s Anxiety Inventory

**Fig. 1:**
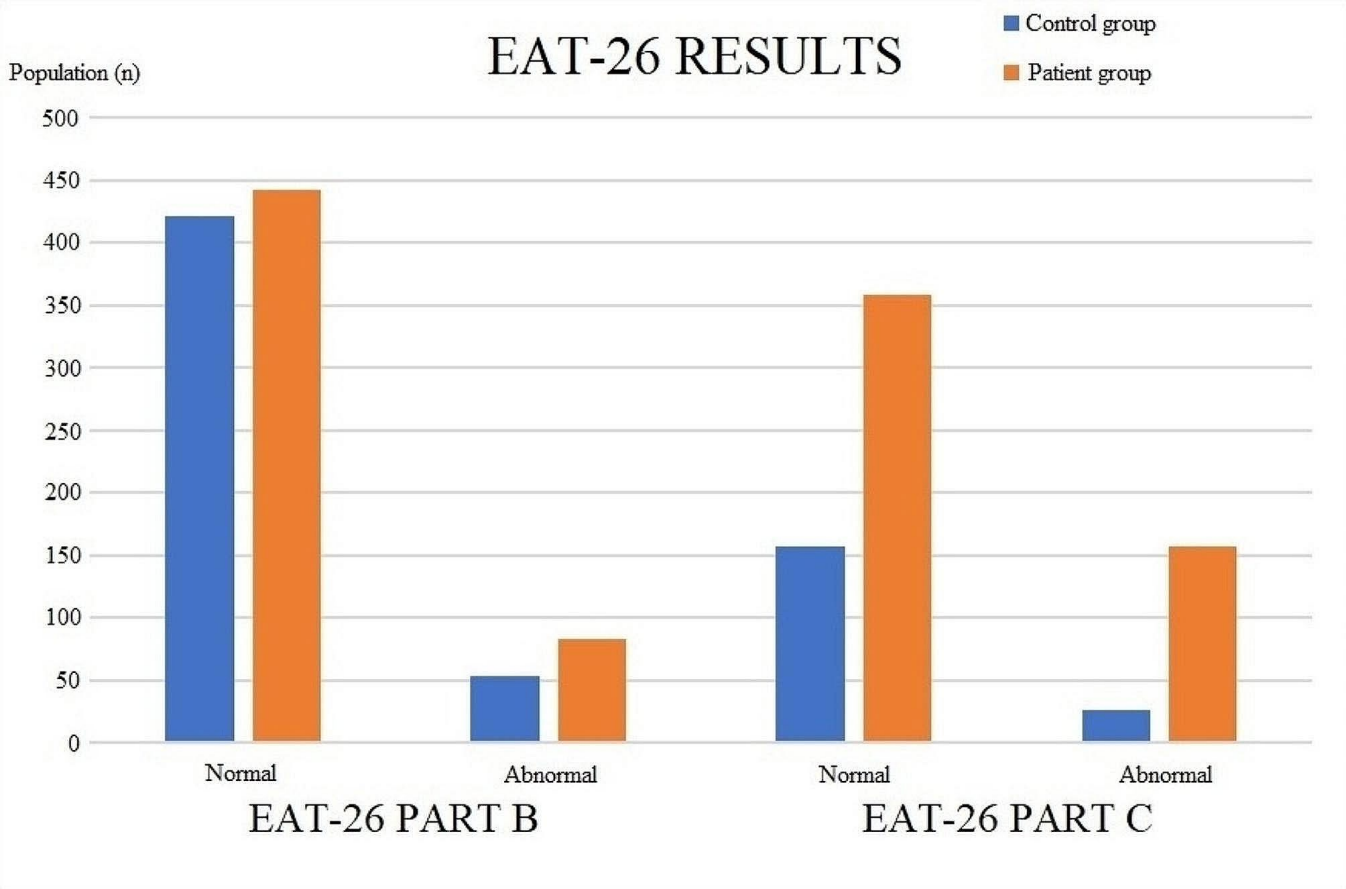
EAT-26 results of the study population

EAT-26 results of the study population were evaluated. 83 participants (15.6%) in control group had abnormal EAT-26 part B scores whereas 86 patients (16.2%) in patient group had abnormal EAT-26 part B scores. 77 participants (14.5%) in control group had abnormal EAT-26 part C scores whereas 166 patients (31.2%) in patient group had abnormal EAT-26 part C scores. Patients with migraine had higher rates of abnormal eating habits compared to control group for both parts B and C of the test and these differences were both statistically significant. There was not a statistically significant difference in terms of eating habits when patients were compared amongst themselves with regard to having migraine with or without aura. Part A of EAT-26 test revelaed no statistically significant difference between two groups. The participants with abnormal eating habits in part C were referred to a psychiatrist. EAT-26 results of the study population are given in Table 4; Fig. 1.

**Table 4:** EAT-26 results of the study population

	Control group (*n* = 531)		Patient group (*n* = 531)		
	n	%	n	%	p
EAT-26 Part B	531		531		
Normal	448	84.4	445	83.8	0.034
Abnormal	83	15.6	86	16.2	
EAT-26 Part C	531		531		
Normal	454	85.5	365	69.8	0.0001
Abnormal	77	14.5	166	31.2	

EAT-26: Eating Attitudes Test-26

## Discussion

Migraine is a common disease in the population. The disease is multifactorial in etiology and several triggers are defined causing attacks during disease course. Various food are thought to be causative in migraine attacks although most of these data are not backed by strong evidence in the literature. In addition, eating attitudes have also been investigated in this patient group with no consistent results. It would not be surprising to find a relationship between migraine and EDs, given the fact that migraine is related to psychiatric disorders. Revealing a potential relationship between EDs and migraine would shed light on disease pathogenesis and avail new therapeutic options both in preventive and treatment aspects. This study was designed to investigate the aforementioned proposed link between eating disorders and migraine.

Migraine and EDs share many aspects in demographic parameters, psychopathological background and treatment modalities. 5-Hydroxytriptamine (5-HT) is used for both disease entities which is a data supportive of a common pathophysiologic mechanism. Results from a study revealed higher rates of perfectionism, ineffectiveness, dissatisfaction with body morphology and interpersonal distrust among female migraine patients; backing the potential effect of 5-HT in migraine and EDs. A significant proportion of migraine patients in this study were found to also have higher rates of binge eating, abnormal eating habits and self-vomiting [26].

De Olivera-Souza and colleagues investigated eating habits in young female migraine patients and found that EDs are more common in this group. This study revealed a 1.85 fold increased risk of having bulimia nervosa symptoms in the mentioned patient population [27].

Attitudes are settled ways of thinking or feeling about something. Behaviours can be reflective of attitudes. EDs can be defined by EAT-26. With this index, we evaluated the abnormal eating attitudes in migraine patients. Abnormal eating attitudes lead to EDs. Stemming from this knowledge, we can plan interventions to improve patients’ eating attitudes and lower the social pressure put on them. By knowing patients’ attitudes towards food consumption, we can estimate their behaviours and take therapeutical actions against behaviours that have negative impact on patients’ well beings [28, 29].

Our study results indeed reflected the abnormal eating attitudes in migraine patients. 83 participants (15.6%) in control group had abnormal EAT-26 part B scores whereas 86 patients (16.2%) in patient group had abnormal EAT-26 part B scores. 77 participants (14.5%) in control group had abnormal EAT-26 part C scores whereas 166 patients (31.2%) in patient group had abnormal EAT-26 part C scores. Patients with migraine had higher rates of abnormal eating habits compared to control group for both parts B and C of the test and these differences were both statistically significant. The participants with abnormal eating habits in part C were referred to a psychiatrist.

Another study also revealed the relationship of EDs with migraine. Anxiety, depression, disability and quality of life were found to be negatively correlated to EDs in migraine patients compared to migraine patients with normal eating attitudes. Migraine related disability and anxiety were found to be higher in migraine patients with EDs. Neuropsychiatry based biological studies would better reveal this multifaceted relationship [30].

Contrary to these studies, Seidel et al. found no correlation between migraine prevalance and EDs [31]. Studies investigating migraine and ED relationship are low in patient population numbers, hence no strong evidence could be revealed to this date. This study is a multi-center, prospective study with a large population both in migraine patients and control group. Study population was selected from a large pool of participants with similar characteristics in terms of parameters like sex, age, body-mass index.

Female migraine patients were found to have higher prevalance of EDs, specifically anorexia nervosa and bulimia nervosa, compared to males in a study by D’Andrea and colleagues. Our study also revealed the female predominance in migraine patients, as females were higher in percentage for both patient and control groups in the study (69.5% and 60.8% respectively). Migraine and EDs were found to have similar metabolic and physiologic derangements. Migraine and other headache subtypes such as tension headache were also compared and close to 75% of migraine patients were found to have EDs. Serum tyramine (Tyr) and dopamine (DA) levels were found to be high and noradrenaline (NE) levels were found to be low in this study. The alterations in levels of these biochemical compounds are suggestive of abnormal hypothalamic and limbic circuitries in ED pathogenesis [32].

Serotonin (5-HT) is a neurotransmitter found in synapses of neurons. It is a commonly found monoamine in neurologic system as well as other organs. Serotonin plays roles in migraine, anxiety, depression, EDs, systemic and pulmonary hypertension, irritable bowel syndrome pathogenesis. Studies revealed mechanism serotonin partakes in disease pathogenesis of both migraine and EDs [12]. Topiramate was also found to be related to EDs in migraine patients, which is reflective of the importance of drug selection in migraine patients [33].

A large study conducted by Hommer et al. on adolescents compared various mental disorders with the presence or absence of headache subtypes. EDs were also included in mental disorders category in this study, with binge eating comprising a large proportion of EDs over anorexia nervosa and bulimia nervosa. The study revealed that EDs are positively correlated with headaches, albeit migraine patients had a lower rate of EDs compared to other headache types [34].

Wang et al. carried out a large cohort study which investigated children to mothers with migraine and without migraine in terms of development of psychiatric disorders. Children born from 1978 to2012 were included in the cohort and the median follow-up time was 19 years. with a median follow-up time of 19 years. Migraine in mothers were correlated to psychiatric disorders in study population, although children born to mothers with migraine were not found to be at increased risk of ED development [35].

Our study results revealed higher scores of BDI and BAI in migraine patients with statistical significance. 18 participants (3.4%) in control group had severe scores in BDI whereas 31 patients (5.8%) in patient group had severe scores in BDI. In temrs of BAI, 38 participants (7.2%) in control group had severe scores in BAI whereas 75 patients (14.1%) in patient group had severe scores in BAI. This data is in line of a review by George and colleagues which mentioned similar results in terms of migraine and psychiatric disorder association [36].

Another study which was carried out by Mustelin et al. found that relatives of migraine patients with depression were to have higher rates of EDs risk throughout their lifespan. Coexistence of depression with migraine was emphasized in this study [37].

Literature data has shown that the common pathogenetic pathway in migraine and EDs is 5-HT. This common pathway is important in that it requires the usage of similar medications in both disorders. Observation of these patients during and after therapy is also important in disease management. Psychiatric disorders are common in migraine patients. Modification of lifestyle and biobehavioral therapies are shown to be effective in this patient group. Patients on multiple medications and with contraindications to drug therapy are candidates for cognitive behavioral therapy (CBT). CBT is shown to be effective in multiple psychiatric disorders. It is also effective on attitudes and behaviours. Studies revealed the efficacy of CBT on eating attitudes and EDs [38, 39]. CBT on children with EDs was found to be effective against depression and helpful with weight loss [40].

There are strengths and limitations of our study. This study is a prospective, multi-center in adult migraine patients with a large study population. An important strength of this study is the prospective and multi-center nature of design. Enrolling a large number of patients from different parts of Turkey resulted in a more heterogenous study population, which better reflects general population. Another strength of the study was exclusion of patients with a prior diagnosis of psychiatric disorder, which prevented a bias in test results applied in the study. Results of the study revealed that eating attitude disorders, depression and anxiety are more common in migraine patients compared to control group. Depression and anxiety both can affect eating attitudes, which can be mentioned as a limitation of the study. Comparison of the study results within different migraine subtypes would also have yielded important results, which is another limitation of the study. Studies which will be planned in the future with more parameters including patients with various psychiatric disorders would reveal more about the interactions between these disease entities. Determination of potential modifiable factors are important in disease management, both for planning of therapeutical interventions and understanding a common pathogenesis for future studies.

In conclusion, migraine and EDs have similar patient characteristics, suggestive of a common disease pathogenesis. In this study, migraine patients were found to have higher rates of EDs, as well as depression and anxiety symptoms.

## Data Availability

We can provide our Excel and SPSS data files upon request.

## Abbreviations

ICHD-3: The International Classification of Headache Disorders-3
ED: Eating Disorder
BN: Bulimia Nervosa
AN: Anorexia Nervosa
CFT: Compassion-Focused Therapy
EAT-26: Eating Attitudes Test-26
BDI: Beck’s Depression Inventory
BAI: Beck’s Anxiety Inventory
BMI: Body-Mass Index
5-HT: 5-Hydroxytriptamine
CBT: Cognitive Behavioral Therapy
VAS: Visual Analogue Scale
